# Interpreting a standardized and normalized measure of neighborhood socioeconomic status for a better understanding of health differences

**DOI:** 10.1186/s13690-021-00750-w

**Published:** 2021-12-15

**Authors:** Masayoshi Oka

**Affiliations:** Department of Management, Faculty of Management, Josai University, 1-1 Keyakidai, Sakado City, Saitama Prefecture 350-0295 Japan

**Keywords:** Standardization, Normalization, Neighborhood socioeconomic status, Multilevel analysis

## Abstract

**Background:**

Standardization and normalization of continuous covariates are used to ease the interpretation of regression coefficients. Although these scaling techniques serve different purposes, they are sometimes used interchangeably or confused for one another. Therefore, the objective of this study is to demonstrate how these scaling techniques lead to different interpretations of the regression coefficient in multilevel logistic regression analyses.

**Methods:**

Area-based socioeconomic data at the census tract level were obtained from the 2015–2019 American Community Survey for creating two measures of neighborhood socioeconomic status (SES), and a hypothetical data on health condition (favorable versus unfavorable) was constructed to represent 3000 individuals living across 300 census tracts (i.e., neighborhoods). Two measures of neighborhood SES were standardized by subtracting its mean and dividing by its standard deviation (SD) or by dividing by its interquartile range (IQR), and were normalized into a range between 0 and 1. Then, four separate multilevel logistic regression analyses were conducted to assess the association between neighborhood SES and health condition.

**Results:**

Based on standardized measures, the odds of having unfavorable health condition was roughly 1.34 times higher for a one-SD change or a one-IQR change in neighborhood SES; these reflect a health difference of individuals living in relatively high SES (relatively affluent) neighborhoods and those living in relatively low SES (relatively deprived) neighborhoods. On the other hand, when these standardized measures were replaced by its respective normalized measures, the odds of having unfavorable health condition was roughly 3.48 times higher for a full unit change in neighborhood SES; these reflect a health difference of individuals living in highest SES (most affluent) neighborhoods and those living in lowest SES (most deprived) neighborhoods.

**Conclusion:**

Multilevel logistic regression analyses using standardized and normalized measures of neighborhood SES lead to different interpretations of the effect of neighborhood SES on health. Since both measures are valuable in their own right, interpreting a standardized and normalized measure of neighborhood SES will allow us to gain a more rounded view of the health differences of individuals along the gradient of neighborhood SES in a certain geographic location as well as across different geographic locations.

## Background

Theoretical and empirical foundations of neighborhoods and health research provide a strong basis to conclude that "where we live" matters for our health, over and above "who we are" [1, 2]. Among the various neighborhood characteristics examined in the United States (US) and other countries, review articles show that neighborhood socioeconomic status (SES) has been consistently associated with risky health behaviors and unfavorable health outcomes [3–5], cardiovascular disease [6], coronary heart disease [7], depression [8, 9], mortality [10], and obesity and physical inactivity [11–13], after adjusting for (accounting for) individual socio-demographic characteristics (e.g., age, gender, race/ethnicity, educational attainment, income level, marital status, and/or occupational category). Note that neighborhood SES has also been referred to as neighborhood deprivation, neighborhood socioeconomic advantage, neighborhood socioeconomic condition, or neighborhood socioeconomic position in these previous studies; although a particular terminology has been favored by different authors, these terms generally refer to a change from the affluent to deprived neighborhoods (or vice versa) in a given study area. By and large, multilevel logistic regression models [14–17] have been used in these previous studies to avoid individualistic and ecological fallacies [18–20].

While a large number of studies have been conducted to date [3–13], some authors argued that the degree of association between neighborhood SES and health has often been modest [3–5]. One possible explanation for such critics is that a continuous measure of neighborhood SES has not been scaled to reflect a change from the highest to lowest neighborhood SES (i.e., the most affluent to most deprived neighborhoods). In regression analyses, for instance, continuous covariates (x) are usually standardized by subtracting its mean ($\overline{\mathrm{x}}$) and then dividing by its standard deviation (SD): (x – $\overline{\mathrm{x}}$) / SD [21]. This ensures a standard normal distribution with a mean of 0 and a SD of 1, and thus the exponentiated regression coefficients in multilevel logistic regression analyses represent the changes in the odds of an event for a one-SD change in x. If continuous covariates are skewed, they are usually divided by its interquartile range (IQR): x / IQR [22]. This is a common trick used in air pollution epidemiology [23–28]. The IQR is defined as the distance between the 25th and 75th percentiles of a distribution, and thus the exponentiated regression coefficients in multilevel logistic regression analyses represent the changes in the odds of an event for a one-IQR change in x. Since the IQR is a robust estimate of SD [29], this IQR scaling provides a useful comparison between two locations on a distribution regardless of the degree of skewness.

Standardizing continuous covariates are a common practice in quantitative research to provide a consistent metric for assessing the relative degree of regression coefficients. However, this standardized degree of relationships lacks intuitive meaning outside the realm of statistical analysis (i.e., what is a one-SD change or a one-IQR change in real-life situations?). To improve the interpretability, regression coefficients showing not only a typical deviation from the center (i.e., the mean or the median), but also a deviation between both ends of the spectrum provide a more rounded view of the extent of health differences. To this end, continuous covariates can be normalized into a range between 0 and 1: (x – x_min_) / (x_max_ – x_min_) [21]. Note that "standardization" of continuous covariates generally refers to subtracting the mean and scaling by the SD or to scaling by the IQR, whereas "normalization" refers to scaling into the range between 0 and 1. Unlike the standardized covariates, the exponentiated regression coefficients of normalized covariates in multilevel logistic regression analyses now represent the changes in the odds of an event from the lowest value to the highest value in x. For these reasons, the degree of association between neighborhood SES and health may appear modest in previous studies [3–5] because a continuous measure of neighborhood SES has not been usually normalized before incorporating into multilevel logistic regression models.

Standardization and normalization of continuous covariates are used to ease the interpretation of regression coefficients. In the context of neighborhoods and health research [1, 2], for example, a standardized measure of neighborhood SES is suited for interpreting health differences of individuals living in relatively high SES (relatively affluent) neighborhoods and those living in relatively low SES (relatively deprived) neighborhoods; on the other hand, a normalized measures of neighborhood SES is suited for interpreting health differences of individuals living in highest SES (most affluent) neighborhoods and those living in lowest SES (most deprived) neighborhoods. Although these scaling techniques serve different purposes, they are sometimes used interchangeably or confused for one another. To avoid ambiguity in the future, the objective of this paper is to show how using a standardized and normalized measure of neighborhood SES leads to different interpretations of the regression coefficient in multilevel logistic regression analyses. Using St. Louis, Missouri (MO) as the study area, a serious of simulation analyses were conducted as an example to illustrate this point, and some methodological considerations are discussed to improve the quality of research synthesis for translating and disseminating research findings to practice.

## Methods

St. Louis, MO is located in the Midwestern part of the US, and approximately 480 km southwest of Chicago, Illinois (the largest city in the Midwestern US). The city core is situated on the Mississippi Riverfront in St. Louis City (which functions as its own county), but vibrant urban neighborhoods also exist in its adjacent St. Louis County and peripheral St. Charles County. Since these three urban counties embrace most of the local urban amenities, a combination of St. Louis City, St. Louis County, and St. Charles County was used to refer to "St. Louis, MO" in this study, and was used to define a geographic boundary of the study area. By including periurban areas (i.e., suburban areas surrounding the urban area), which are an important source of annexation for understanding the health of city dwellers [30], the study area encompasses a total land area of about 3000 km^2^ with a little over 1.5 million population.

For simulating a study of neighborhood effect on health in St. Louis, MO (the study area), which is built upon the conceptual and methodological foundations of neighborhoods and health research [1, 2], a hypothetical data was constructed to represent 3000 individuals (Table 1). Here, a binary measure was used to denote "health condition" (the outcome of interest) in which 2100 individuals (70%) were coded as “favorable” and remaining 900 individuals (30%) were coded as “unfavorable.” In addition, one continuous and three categorical measures were constructed in efforts to portray individuals’ socio-demographic characteristics (e.g., age, gender, race/ethnicity, educational attainment, income level, marital status, and/or occupational category). Treating this hypothetical data as individual-level data, conceptual and methodological approaches for conducting a series of simulation analyses implemented in this study are described below.

**Table 1:** Description of a hypothetical data (3000 individuals)

**Binary Outcome of Interest**	
Health Condition	
Favorable	70.0%
Unfavorable	30.0%
**Individual-level Characteristics**	
Trait 1	
Minimum	25.0
Mean	42.1
Median	41.0
Maximum	65.0
Trait 2	
Group 1	50.0%
Group 2	50.0%
Trait 3	
Group 1	30.5%
Group 2	28.2%
Group 3	30.8%
Group 4	10.5%
Trait 4	
Group 1	13.5%
Group 2	20.2%
Group 3	23.6%
Group 4	29.4%
Group 5	13.4%

In the study of neighborhood effects on health [1–13], a measure of neighborhood SES has been used to capture the degree of social and material deprivation of a neighborhood, where "deprivation" refers to as a state of disadvantage below socially acceptable degrees compared to its surroundings in a society [31]. To capture the gradient of neighborhood SES in St. Louis, MO, area-based socioeconomic data at the census tract level were obtained from the 2015–2019 American Community Survey (ACS). Note that the ACS is an ongoing national survey conducted by the US Census Bureau since 2005, and is the primary source of demographic, socioeconomic, and housing information that replaced the long form of the US decennial census. The five-year ACS estimates are based on a larger sample size, and thus more accurate than the one- and three-year ACS estimates. See Herman [32] for background information about the ACS. Census tracts has been used to denote "neighborhoods" in US studies [3–13] because they are a national creation of democratic governance informed by local inputs, and are also historically in accordance with uniform standards [33].

Among the various ways in which a neighborhood SES has been measured in US studies [3–13], a composite measure of neighborhood SES (which is based on a combination of multiple area-based socioeconomic indicators) was derived from the index of socioeconomic position (SEP) developed by Krieger et al. [34]. SEP is a summary score constructed by summing the Z-scores of median household income (US $), owner-occupied homes worth ≥$300,000 (%), population in working class (%), population with less than high school education (%), and population below the poverty line (%). Recently, Oka [35] suggested that median household income (MHI) or median family income (MFI) may be used to capture the gradient of neighborhood SES in a given study area instead of a composite measure. Therefore, SEP and MHI were used as measures of neighborhood SES to show common measurement techniques used in neighborhoods and health research.

**Fig. 1:**
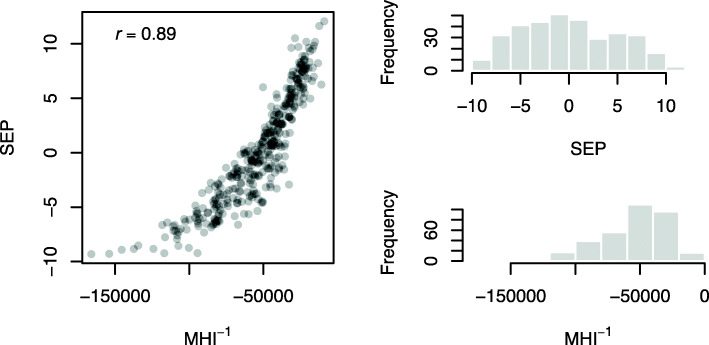
Correlation plot and histogram of two measures of neighborhood socioeconomic status in St. Louis, MO (382 census tracts). SEP = a composite measure of socioeconomic position [34]. MHI^− 1^ = median household income divided by − 1 [35]

By definition, an increase in MHI and MFI correspond to a change from deprived neighborhoods to affluent neighborhoods in a given area. In order to reflect a change from affluent neighborhoods to deprived neighborhoods, MHI and MFI can be divided by − 1, then be denoted as MHI^− 1^ and MFI^− 1^ [35]. Note that reversing the direction of MHI and MFI do not affect the width and height of distribution. Using the Pearson product-moment correlation coefficient (*r*) as a statistical test, MHI^− 1^ and MFI^− 1^ was strongly and positively correlated with each other (*r* = 0.93) and SEP and MHI^− 1^ was also strongly and positively correlated with each other (*r* = 0.89). Correlation plot and histogram of SEP and MHI^− 1^ are shown in Fig. 1. In this study, SEP followed an approximately normal (bell-shaped or Gaussian) distribution, and thus was standardized by subtracting its mean and then dividing by its SD. Since MHI^− 1^ followed a slightly skewed distribution, it was standardized by dividing by its IQR. Regardless of the shape of distributions, these two measures of neighborhood SES were normalized into the range between 0 and 1. Standardization and normalization of SEP and MHI^− 1^ were based on 382 census tracts that lie within St. Louis, MO (two out of 384 census tracts were omitted due to missingness of area-based socioeconomic indicators in the 2015–2019 ACS data).

As an analytical note, skewness of neighborhood-level covariate(s) may pose challenges when an outcome of interest is a continuous measure (e.g., body mass index, BMI). Without due consideration, the assumption of linearity, normality, and/or homoscedasticity of residuals may be violated [36–38]. While power, square root, log, and other types of transformations are commonly applied to skewed continuous covariates for satisfying the key assumptions, covariate transformations modify the measurement scale, alter the curvilinear relationship, and obscure the results of regression analysis [39]. Taking these under consideration, MHI^− 1^ was not transformed prior to the aforementioned standardizing and normalizing processes.

Unlike multilevel linear regression models, multilevel logistic regression models have less stringent requirements [14–17]. In previous studies [3–13], an outcome of interest has often relied on or classified into a binary measure (e.g., BMI < 30 versus BMI ≥30). For this reason, as long as the linearity of the logit assumption is satisfied, multilevel logistic regression models can be used for examining an association between a slightly skewed measure of neighborhood SES and a health-related outcome of interest after adjusting for (accounting for) individual-level sociodemographic characteristics.

To quantitatively demonstrate how using standardized and normalized measures of neighborhood SES lead to different interpretations of the regression coefficient, a hypothetical data of 3000 individuals (Table 1) was allocated across 300 neighborhoods (i.e., census tracts). In other words, four measures of neighborhood SES and a hypothetical data of 3000 individuals were combined into one dataset. Then, a series of multilevel logistic regression analyses was carried out to examine the association between neighborhood SES and health condition (Table 2). Note that these simulation analyses are based on a two-level model where individuals (at the first level) are nested within neighborhoods (at the second level).

**Table 2:** Simulation analyses of the association between neighborhood socioeconomic status and health condition in St. Louis, MO (3000 individuals across 300 census tracts)*

		Model 1	Model 2	Model 3	Model 4
**Fixed Effects**		OR (95% CI)	OR (95% CI)	OR (95% CI)	OR (95% CI)
*Neighborhood-level Covariates*					
SEP	(standardized)	1.32 (1.17, 1.48)			
MHI^−1^	(standardized)		1.35 (1.14, 1.60)		
SEP	(normalized)			3.26 (1.93, 5.50)	
MHI^−1^	(normalized)				3.70 (1.78, 7.70)
*Individual-level Covariates*					
Trait 1		1.03 (1.02, 1.04)	1.03 (1.02, 1.04)	1.03 (1.02, 1.04)	1.03 (1.02, 1.04)
Trait 2	Group 1	Ref.	Ref.	Ref.	Ref.
	Group 2	1.22 (1.02, 1.45)	1.22 (1.03, 1.46)	1.22 (1.02, 1.45)	1.22 (1.03, 1.46)
Trait 3	Group 1	Ref.	Ref.	Ref.	Ref.
	Group 2	1.20 (0.94, 1.52)	1.21 (0.95, 1.54)	1.20 (0.94, 1.52)	1.21 (0.95, 1.54)
	Group 3	1.24 (0.97, 1.57)	1.27 (1.00, 1.61)	1.24 (0.97, 1.57)	1.27 (1.00, 1.61)
	Group 4	1.43 (1.03, 1.98)	1.49 (1.07, 2.05)	1.43 (1.03, 1.98)	1.49 (1.07, 2.05)
Trait 4	Group 1	Ref.	Ref.	Ref.	Ref.
	Group 2	1.10 (0.80, 1.52)	1.10 (0.80, 1.52)	1.10 (0.80, 1.52)	1.10 (0.80, 1.52)
	Group 3	1.24 (0.91, 1.71)	1.25 (0.91, 1.72)	1.24 (0.91, 1.71)	1.25 (0.91, 1.72)
	Group 4	1.19 (0.86, 1.64)	1.24 (0.90, 1.70)	1.19 (0.86, 1.64)	1.24 (0.90, 1.70)
	Group 5	1.38 (0.95, 2.02)	1.47 (1.01, 2.14)	1.38 (0.95, 2.02)	1.47 (1.01, 2.14)
**Random Effects**		Variance (SE)	Variance (SE)	Variance (SE)	Variance (SE)
Census Tract		0.257 (0.507)	0.276 (0.525)	0.257 (0.507)	0.276 (0.525)
**Goodness-of-Fit Measures**					
Akaike information criterion		3527.677	3534.992	3527.677	3534.992
Bayesian information criterion		3599.753	3607.069	3599.753	3607.069
Log-Likelihood		− 1751.838	− 1755.496	−1751.838	−1755.496
Deviance		3503.677	3510.992	3503.677	3510.992

*When analyzing the actual health data, multilevel models must adjust for (account for) individual-level socio-demographic characteristics (e.g., age, gender, race/ethnicity, educational attainment, income level, marital status, and/or occupational category)

*Ref*. reference group

*SE* standard error

*OR* odds ratio

*CI* confidence interval

*SEP* a composite measure of socioeconomic position [34]

*MHI*^*− 1*^ median household income divided by − 1 [35]

Computation, standardization and normalization of SEP and MHI^− 1^, as well as a series of simulation analyses were carried in R version 4.0.5 [40]. The glmer function in the lme4 package [41] was used for conducting a series of multilevel logistic regression analyses.

## Results

Four separate multilevel logistic regression analyses were conducted to simulate how using standardized and normalized measures of SEP and MHI^− 1^ could lead to different interpretations of the association between neighborhood SES and health condition (Table 2). Based on standardized measures, holding all individual-level covariates constant, the odds of having unfavorable health condition was roughly 1.34 times higher for a one-SD change or a one-IQR change in neighborhood SES (OR = 1.32, 95% CI = 1.17–1.48 in Model 1 and OR = 1.35, 95% CI = 1.14–1.60 in Model 2); these reflect a health difference of individuals living in relatively high SES (relatively affluent) neighborhoods and those living in relatively low SES (relatively deprived) neighborhoods. On the other hand, when these standardized measures were replaced by its respective normalized measure, keeping all individual-level covariates held constant, the odds of having unfavorable health condition was roughly 3.48 times higher for a full unit change in neighborhood SES (OR = 3.26, 95% CI = 1.93–5.50 in Model 3 and OR = 3.70, 95% CI = 1.78–7.70 in Model 4); these reflect a health difference of individuals living in highest SES (most affluent) neighborhoods and those living in lowest SES (most deprived) neighborhoods. These results corroborate Oka’s [35] suggestion that MHI^− 1^ provides the same explanatory power as a composite measure of neighborhood SES (i.e., SEP in this study). While results shown in Table 2 must not be considered as empirical evidence, a comparison of Models 1 and 2 with Models 3 and 4, respectively, also highlights how scaling techniques (i.e., standardization and normalization) lead to different interpretations of the association between neighborhood SES and health condition.

When comparing four multilevel logistic regression in Table 2, it is important to point out that the estimation of individual-level fixed effects, random effects, and goodness-of-fit measures were exactly the same between models using the standardized and normalized measure of SEP and MHI^− 1^ (Model 1 versus Model 3 and Model 2 versus Model 4). Therefore, Table 2 underscores the fact [21, 22] that the interpretation of the association between neighborhood SES and health condition is sensitive to how SEP and MHI^− 1^ were scaled, but replacing standardized measures with its respective normalized measure do not influence other parameter estimates (although negligible differences may occur due to rounding errors).

As noted above, standardized and normalized measures are used in regression analyses for different purposes. However, what seems to have only a modest effect on health condition may turn out to have a much larger effect, depending on how the measure of neighborhood SES was scaled (Table 2). While a hypothetical data on health condition was used as an example in this study, the same notion of analogy can be used for empirical studies on various health behaviors and health outcomes. Hence, the degree of association between neighborhood SES and health must be interpreted with caution when compiling and/or synthesizing research findings.

## Discussion

In the study of neighborhood effects on health [1–13], one of the important objectives of the research is to understand the health differences of individuals living in highest SES (most affluent) neighborhoods and those living in lowest SES (most deprived) neighborhoods in a certain geographic location as well as across different geographic locations. Therefore, a normalized measure of neighborhood SES can be used in this regard. However, there are arguably different perspectives toward capturing the gradient of neighborhood SES in a given study area (i.e., whether to use SEP or MHI^− 1^) and/or presenting results from a multilevel regression analysis in a more intuitive manner (i.e., whether to convert a continuous measure into categories or not) that may arise during the research process. In order to foster informative research, practical arguments against using SEP and categorizing MHI^− 1^ are discussed below.

First, using MHI^− 1^ is recommended over SEP (or any other composite measure of neighborhood SES) to avoid the influence of outliers (or extreme values). In quantitative research, the presence of outliers (or an outlier) can bias the results of statistical analyses [42–44]. Among the various analytical implications [42–44], outliers would strongly distort the mean and increase the SD and range, but generally not the median and IQR. Even a single outlier has a substantial influence on the results of a regression analysis [42]. Therefore, retaining outliers in SEP (or any other composite measure of neighborhood SES) disproportionately magnifies the association between neighborhood SES and health, particularly when a normalized measure is used. To avoid any misleading results from a regression analysis, graphical tools (e.g., boxplots and histograms) are commonly used for screening influential outliers as a part of explanatory data analysis. Unlike the univariate cases (e.g., MHI^− 1^), however, detecting and removing outliers is not a trivial task in the multivariate cases (e.g., SEP). That is, a multivariate outlier need not be an outlier when considering each variable separately. Note that SEP is a summary score derived by summing the Z-scores of six area-based socioeconomic indicators [34], which is a technique used for handling multivariate data. For these reasons, removing outliers in MHI^− 1^ and then standardizing or normalizing it prior to conducting a multilevel regression analysis is likely to provide an accurate estimation of the association between neighborhood SES and health.

Second, using a continuous measure of MHI^− 1^ is recommended over its alternative categorized measures to prevent the loss of information and statistical power. In regression analyses, continuous measures are frequently converted into multiple categories to illustrate the association between a continuous measure of exposure and a health-related outcome [45–47]. For example, tertiles (3-quantiles), quartiles (4-quantiles), and quintiles (5-quantiles) are used to split a continuous measure into three, four, and five categories of equal size, respectively. The underlying motivation for categorizing continuous measures is not only to present an intuitive presentation of the exposure-response relation, but also to avoid the well-known problems (e.g., loss of information and statistical power) associated with dichotomizing (i.e., mean or median splitting) continuous measures [48–50]. While categorization of continuous measures can be much more effective than dichotomization, a loss of efficiency attributed to inadequate assumptions about within-category heterogeneity and between-category differences is quite substantial [46, 47]. Therefore, categorization of continuous measures is strongly discouraged in regression analyses [51, 52]. See Bennette and Vickers [52] for a useful discussion on this topic. Taking these points into account, using a continuous measure of MHI^− 1^ is likely to provide an accurate estimation of the association between neighborhood SES and health.

Notwithstanding the usefulness of standardized and normalized MHI^− 1^, technical considerations warrant attention in the model building process. In the study of neighborhood SES and health, standardization of MHI^− 1^ by its IQR, following Babyak’s [22] suggestion, may be sufficient if only the effect of neighborhood SES is of interest, but may be insufficient when the effects of other neighborhood characteristics are also of interest (i.e., two or more neighborhood-level covariates in the same multilevel regression model). Particularly in obesity research, review articles showed that population density (or residential density), which is a measure of neighborhood urbanness [53], has been consistently associated with higher amounts of physical activity [54, 55]. To bring measures of neighborhood SES and neighborhood urbanness into proportion with one another, for example, they need to be centered at zero by subtracting its median ($\overset{\sim }{\mathrm{x}}$) and then dividing by its IQR: (x – $\overset{\sim }{\mathrm{x}}$) / IQR. Median-centering is particularly important when interaction between neighborhood-level covariates or between neighborhood- and individual-level covariates are examined [56]. In the absence of interaction term(s), median-centering of standardized measures would be unnecessary. Therefore, careful consideration is required in the model building process.

As a final remark, there are two supplementary scaling techniques worth mentioning. For one, continuous covariates can also be standardized by subtracting its mean and then dividing by two times its SD: (x – $\overline{\mathrm{x}}$) / 2*SD [57]. This allows resulting regression coefficients to be directly comparable to binary covariate(s) in the same regression model. For another, continuous covariates can also be normalized into a range between − 1 and + 1: 2*[(x – x_min_) / (x_max_ – x_min_)] – 1 [58]. By centering at zero, it prevents over- or under-estimating (i.e., magnifying or mitigating) the intercept term of regression models, which would be particularly important in (multilevel) linear regression analyses. While these scaling techniques [57, 58] are applicable for continuous covariates following a normal (bell-shaped or Gaussian) distribution, not for those following a skewed distribution, both provide a means to ease the interpretation of regression coefficients without any harm on other parameter estimates. Hence, in addition to the analytical benefits of most common and widely used scaling techniques [21, 22] described, demonstrated, and discussed above, these two supplementary scaling techniques [57, 58] may be equally beneficial for neighborhoods and health research.

## Conclusion

Interpretation of an association between neighborhood SES and health is sensitive to how a measure of neighborhood SES is scaled. As demonstrated by a series of simulation analyses (Table 2), using standardized and normalized measures of neighborhood SES led to different interpretations of the effect of neighborhood SES on health condition (favorable versus unfavorable). These suggest that the “evidence” for modest effects of neighborhood SES on health [3–5] may be misleading or deceptive. A degree of association may appear weak (small), modest (medium), or strong (large) depending on how a measure of neighborhood SES has been scaled (or even categorized) in previous studies. Every so often, a scaling process has not been clearly described in scientific articles and reports (i.e., it is “hidden” under the degree of association). Therefore, we must be transparent and cautious about the scaling technique used in multilevel logistic regression analyses, as well as in multilevel linear regression analyses, upon interpreting the regression coefficients.

The key takeaway from this study is that the analytical benefits of most common and widely used scaling techniques (i.e., standardization and normalization) [21, 22] collectively provide a better understanding of the relationship between neighborhood SES and health. Viewing a measure of neighborhood SES as "a neighborhood affluence-deprivation continuum," the term used by Oka [35], health differences of individuals living in highest SES (most affluence) neighborhoods and those living in lowest SES (most deprived) neighborhoods may be quite distinct, but others in between may not be perceptibly different from each other. To gain a more rounded view of the extent of health differences, interpreting not only based on a familiar unit of one-SD or one-IQR change in neighborhood SES, but also based on a full unit change in neighborhood SES would be a necessary course of action. Hence, future studies utilizing two scaling techniques [21, 22] in their multilevel logistic regression analyses (i.e., one model with a standardized measure and another model with a normalized measure) will allow us to gain a better grasp on the health differences of individuals along the gradient of neighborhood SES in a certain geographic location as well as across different geographic locations.

## Data Availability

The datasets used and/or analyzed in this study are available from the author on reasonable request.

## Abbreviations

SES: Socioeconomic Status
SD: Standard Deviation
IQR: Interquartile Range
ACS: American Community Survey
SEP: Socioeconomic Position
MHI: Median Household Income
MFI: Median Family Income
BMI: Body Mass Index
OR: Odds Ratio
CI: Confidence Interval

## References

[1] Kawachi I, Berkman LF. Neighborhoods and health. New York: Oxford University Press; 2003. 10.1093/acprof:oso/9780195138382.001.0001.

[2] Duncan DT, Kawachi I. Neighborhoods and health. 2nd ed. New York: Oxford University Press; 2018. 10.1093/oso/9780190843496.001.0001

[3] Robert SA (1999). Socioeconomic position and health: the independent contribution of community context. Annu Rev Sociol.

[4] Pickett KE, Pearl M (2001). Multilevel analyses of neighborhood socioeconomic context and health outcomes: a critical review. J Epidemiol Community Health.

[5] Riva M, Gauvin L, Barnett TA (2007). Toward the next generation of research into small area effects on health: a synthesis of multilevel investigations published since July 1998. J Epidemiol Community Health.

[6] Diez Roux AV (2003). Residential environments and cardiovascular risk. J Urban Health.

[7] Chaix B (2009). Geographic life environments and coronary heart disease: a literature review, theoretical contributions, methodological updates, and a research agenda. Annu Rev Public Health.

[8] Kim D (2008). Blues from the neighborhood? Neighborhood characteristics and depression. Epidemiol Rev.

[9] Mair C, Diez Roux AV, Galea S (2008). Are neighbourhood characteristics associated with depressive symptoms? A review of evidence. J Epidemiol Community Health.

[10] Meijer M, Röhl J, Bloomfield K, Grittner U (2012). Do neighborhoods affect individual mortality? A systematic review and meta-analysis of multilevel studies. Soc Sci Med.

[11] Booth KM, Pinkston MM, Poston WS (2005). Obesity and the built environment. J Am Diet Assoc.

[12] Papas MA, Alberg AJ, Ewing R, Helzlsouer KJ, Gary TL, Klassen AC (2007). The built environment and obesity. Epidemiol Rev.

[13] Black JL, Macinko J (2008). Neighborhoods and obesity. Nutr Rev.

[14] Raudenbush SW, Bryk AS. Hierarchical linear models in social and behavioral research: applications and data analysis methods. 2nd edition ed. Newbury Park: Sage Publications; 2002. 10.1177/0163278704264049

[15] Gelman A, Hill J (2007). Data analysis using regression and multilevel/hierarchical models.

[16] Hox J (2010). Multilevel analysis: techniques and applications.

[17] Snijders TAB, Bosker RJ (2012). Multilevel analysis: an introduction to basic and advanced multilevel modeling.

[18] Diez Roux AV (1998). Bringing context Back into epidemiology: variables and fallacies in multilevel analysis. Am J Public Health.

[19] Diez Roux AV (2000). Multilevel analysis in public Health Research. Annu Rev Public Health.

[20] Subramanian SV, Jones K, Kaddour A, Krieger N (2009). Revisiting Robinson: the perils of individualistic and ecologic fallacy. Int J Epidemiol.

[21] Milligan GW, Cooper MC (1988). A study of standardization of variables in cluster analysis. J Classif.

[22] Babyak MA, VA ML (2009). Rescaling continuous predictors in regression models. Statistical Tips from the Editors of Psychosomatic Medicine.

[23] Baja ES, Schwartz JD, Wellenius GA, Coull BA, Zanobetti A, Vokonas PS, Suh HH (2010). Traffic-related air pollution and QT interval: modification by diabetes, obesity, and oxidative stress gene polymorphisms in the normative aging study. Environ Health Perspect.

[24] Hoffmann B, Luttmann-Gibson H, Cohen A, Zanobetti A, de Souza C, Foley C, Suh HH, Coull BA, Schwartz J, Mittleman M, Stone P, Horton E, Gold DR (2012). Opposing effects of particle pollution, ozone, and ambient temperature on arterial blood pressure. Environ Health Perspect.

[25] Krall JR, Anderson GB, Dominici F, Bell ML, Peng RD (2013). Short-term exposure to particulate matter constituents and mortality in a National Study of U.S. urban communities. Environ Health Perspect.

[26] Bind M-A, Peters A, Koutrakis P, Coull B, Vokonas P, Schwartz J (2016). Quantile regression analysis of the distributional effects of air pollution on blood pressure, heart rate variability, blood lipids, and biomarkers of inflammation in elderly American men: the normative aging study. Environ Health Perspect.

[27] Hao H, Chang HH, Holmes HA, Mulholland JA, Klein M, Darrow LA, Strickland MJ (2016). Air pollution and preterm birth in the U.S. state of Georgia (2002–2006): associations with concentrations of 11 ambient air pollutants estimated by combining community multiscale air quality model (CMAQ) simulations with stationary monitor measurements. Environ Health Perspect.

[28] von Ehrenstein OS, Heck JE, Park AS, Cockburn M, Escobedo L, Ritz B (2016). In utero and early-life exposure to ambient air toxics and childhood brain tumors: a population-based case–control study in California. USA Environ Health Perspect.

[29] Tukey JW (1977). Exploratory data analysis.

[30] Vlahov D, Galea S (2002). Urbanization, urbanicity, and health. J Urban Health.

[31] Townsend P (1987). Deprivation. J Soc Policy.

[32] Herman E (2008). The American community survey: an introduction to the basics. Gov Inform Q.

[33] Krieger N (2006). A century of census tracts: health & the body politic (1906-2006). J Urban Health.

[34] Krieger N, Chen JT, Waterman PD, Soobader M-J, Subramanian SV, Carson R (2003). Choosing area based socioeconomic measures to monitor social inequalities in low birth weight and childhood lead poisoning: the public health disparities geocoding project (US). J Epidemiol Community Health.

[35] Oka M (2015). Measuring a neighborhood affluence-deprivation continuum in urban settings: descriptive findings from four US cities. Demogr Res.

[36] Osborne JW, Waters E (2002). Four Assumptions Of Multiple Regression That Researchers Should Always Test. Pract Assess Res Eval.

[37] Williams MN, Gómez Grajales CA, Kurkiewicz D (2013). Assumptions of Multiple Regression: Correcting Two Misconceptions. Pract Assess Res Eval.

[38] Osborne JW (2013). Normality of residuals is a continuous variable, and does seem to influence the trustworthiness of confidence intervals: A response to, and appreciation of, Williams, Grajales, and Kurkiewicz (2013). Pract Assess Res Eval.

[39] Osborne JW (2002). Notes on the use of data transformations. Pract Assess Res Eval.

[40] R Core Team (2021). R: A Language and Environment for Statistical Computing. Version 4.1.1 ed.

[41] Bates D, Maechler M, Bolker BM, Walker S (2015). Fitting linear mixed-effects models using lme4. J Stat Softw.

[42] Stevens JP (1984). Outliers and influential data points in regression analysis. Psychol Bull.

[43] Osborne JW, Overbay A (2004). The power of outliers (and why researchers should always check for them). Pract Assess Res Eval.

[44] Aggarwal CC (2013). Outlier Analysis.

[45] Altman DG, Bland JM (1994). Quartiles, quintiles, centiles, and other quantiles. Br Med J.

[46] O’Brien SM (2004). Cutpoint selection for categorizing a continuous predictor. Biometrics..

[47] Gelman A, Park DK. Splitting a predictor at the upper quarter or third and the lower quarter or third. Am Stat. 2008;62(4):1–8. 10.1198/tast.2009.0001

[48] Cohen J (1983). The cost of dichotomization. Appl Psych Meas.

[49] MacCallum RC, Zhang S, Preacher KJ, Rucker DD (2002). On the practice of dichotomization of quantitative variables. Psychol Methods.

[50] Royston P, Altman DG, Sauerbrei W (2006). Dichotomizing continuous predictors in multiple regression: a bad idea. Stat Med.

[51] Weinberg CR (1995). How bad is categorization. Epidemiology..

[52] Bennette C, Vickers A (2012). Against quantiles: categorization of continuous variables in epidemiologic research, and its discontents. BMC Med Res Methodol.

[53] Oka M, Wong DWS (2016). Spatializing area-based measures of neighborhood characteristics for multilevel regression analyses: an areal median filtering approach. J Urban Health.

[54] Ding D, Sallis JF, Kerr J, Lee S, Rosenberg DE (2011). Neighborhood environment and physical activity among youth: a review. Am J Prev Med.

[55] Durand CP, Andalib M, Dunton GF, Wolch J, Pentz MA (2011). A systematic review of built environment factors related to physical activity and obesity risk: implications for smart growth urban planning. Obes Rev.

[56] Schielzeth H (2010). Simple means to improve the interpretability of regression coefficients. Methods Ecol Evol.

[57] Gelman A (2008). Scaling regression inputs by dividing by two standard deviations. Stat Med.

[58] Han J, Kamber M, Pei J (2012). Data Preprocessing. Data Mining: Concepts and Techniques.

